# Free Acids in the Stomach

**Published:** 1906-03-10

**Authors:** 


### Hospital Clinics.

### FREE ACIDS IN THE STOMACH.

In a note upon indicators of free acids in the gastric contents, Dr. Hector Colwell [1] remarks that the two bodies whose detection is a matter of prime importance to the clinical pathologist are hydrochloric and lactic acids. The most scientific and satisfactory way of obtaining a sample of the contents of the stomach is to administer a test meal and subsequently withdraw it by means of a gastric syphon. But such a proceeding is often impossible owing to the repugnance shown by the patient. In such cases it is necessary to rely upon the material vomited spontaneously. In point of practice patients who are subject to frequent vomiting are usually placed upon a strictly milk diet, and thus obviate the difficulty. But it is very necessary to see that the food administered is fresh milk or milk and water, for such articles as alkaline aërated waters will, of course, vitiate the findings.

For the practical detection of free hydrochloric acid the best method is that which involves the use of phloroglucin and vanillin. The reagents necessary are (1) a solution of vanillin in absolute alcohol, .5 gramme to 50 cubic centimetres; (2) a solution of phloroglucin, 1 gramme in 50 cubic centimetres of absolute alcohol. These should be kept separately in stoppered bottles and in the dark. The technique is as follows:—A few drops of the liquid to be examined are filtered into a white porcelain evaporating dish, two or three drops of each of the above reagents are added, and the whole well mixed with a glass rod. The dish with its contents is then set upon a briskly-boiling water-bath. The use of the water-bath is strongly advised, for by this means a certain constancy of temperature is secured for each determination, while over-heating and the brown discolouration liable to accompany it are thus avoided. This discolouration may give the appearance of a reaction even in the absence of bodies which normally react.

If free hydrochloric acid be present there results as the mixture dries a fine pink or red colour, which will vary in intensity with the quantity of free acid present in the solution. The reaction is extremely delicate, and, if watery solutions alone be used, responds to so minute a quantity as two or three drops of a one-thousandth normal solution of hydrochloric acid.

But the reagent is sensitive to other free mineral and organic acids, a point which acquires practical importance from the fact that mixtures containing the subnitrate of bismuth also contain traces of free nitric acid. The colouration does not appear with lactic, acetic, propionic, butyric, valerianic, isovalerianic, or succinic acids, but is produced by oxalic and malonic acids, and by certain oxy-acids such as tartaric, citric, and malic. It is pointed out that while acetic and monochloracetic acids fail to react, dichloracetic and trichloracetic acids do react, the latter powerfully. Practically, therefore, no acid giving the reaction is likely to occur in vomited material except hydrochloric acid, unless the subnitrate of bismuth is being administered medicinally.

Many varieties of invalid diet were investigated with a view to eliminating sources of error in the test. None appeared to exercise the slightest effect upon the reaction unless present in enormous quantity, except non-alkaline beef tea, which, even when filtered and present in quite small amounts, appeared to be powerfully inhibitory. Experiments were also made with common preservatives of milk, such as boric acid and the borates, salicylic acid and the salicylates, and formaldehyde. Boric and salicylic compounds exerted no effect upon the reaction, but formaldehyde even in dilute solution inhibited it. The author considers, however, that in the minute quantities likely to be employed as a trade preservative it is quite inactive.

For lactic acid the most delicate and reliable indicator is an extremely dilute aqueous solution of ferric chloride. A solution of ferric chloride is prepared whose actual strength is not of importance provided the colour be that of pale sherry. The test is performed as follows: About ten cubic centimetres of distilled water are taken, and tinted a very faint yellow colour with the solution of ferric chloride. A few drops of this mixture placed in a porcelain basin will, on the addition of the merest trace of lactic acid, produce a vivid canary-yellow colouration, which on the addition of ammonia becomes reddish. This last reaction serves to distinguish lactic from tartaric and citric acids, for the colour is in the cases of these acids discharged by the addition of ammonia. Acetic acid produces a reddish tinge; propionic, butyric, and valerianic acids yield a yellow colour, but one lacking the brilliancy of the colour given by lactic acid. It is most important in all these colour tests to employ a good light, preferably daylight. A number of other vaunted indicators are considered by the author, but are all pronounced unreliable.

[1] Archives of the Middlesex Hospital, 1905.

